# Continuous Relative Phase Angle and Variability: A Crossover Analysis of Duration and Surface Effects While Long‐Distance Running Over Treadmill and Over‐Ground Running

**DOI:** 10.1155/abb/5338592

**Published:** 2025-12-20

**Authors:** Zaheen Ahmed Iqbal, Indy Man Kit Ho, Daniel Hung-Kay Chow

**Affiliations:** ^1^ Department of Health and Physical Education, The Education University of Hong Kong, Tai Po, New Territories, Hong Kong, ied.edu.hk; ^2^ Department of Physiotherapy, School of Allied Health Sciences, Manav Rachna International Institute of Research and Studies, Faridabad, India; ^3^ Department of Sport and Recreation, Technological and Higher Education Institute of Hong Kong, Chai Wan, Hong Kong, thei.edu.hk

**Keywords:** continuous relative phase angle, coordination, distance running, inertial measurement units, stride-to-stride variability, treadmill and over-ground running

## Abstract

Running coordination, quantified using continuous relative phase (CRP) and its variability, plays a key role in adapting to dynamic environments; however, how these measures behave during long‐distance running on different surfaces remains unclear. This study compared lower‐limb coordination and variability during prolonged running across treadmill and over‐ground, focusing on how surface and duration affect movement patterns in sagittal‐plane. Eleven healthy adults (nine males) completed 31‐min runs at their preferred speed on both surfaces, on separate days, while data were collected using seven Opal Movement Monitoring inertial measurement units. CRP and its variability were examined across two‐time intervals (initial and final 5 min) and two running surfaces, both over the full gait cycle and within the stance and swing phases. Overall, running duration and surface did not significantly affect coordination across the full gait cycle. However, ankle–knee coordination increased in the final 5 min during stance. Surface‐by‐duration interactions were observed in knee–hip and ankle–knee couplings during over‐ground running. During the swing phase, ankle–hip coordination increased in the final 5 min on both surfaces, with additional interactions appearing in ankle–hip coupling during treadmill running. Coordination variability showed no significant differences across the gait cycle or within stance and swing phases. These findings suggest that lower‐limb coordination patterns, rather than variability, are more sensitive to changes in running duration and surface. The results underscore the importance of considering external running conditions when evaluating coordination and optimizing gait performance in biomechanical assessments.

## 1. Introduction

Running is a fundamental activity across sports, involving repetitive multijoint motion that enhances health but also predisposes individuals to overuse injuries through cumulative joint loading [1, 2]. A critical aspect of running biomechanics is variability, which reflects an athlete’s ability to adapt to dynamic conditions [3]. Lower limb joint angles and their variability differ with running duration and surface, with over‐ground running typically showing greater variability than treadmill running [4]. These differences are essential for designing effective training and rehabilitation programs.

In biomechanics, coordination, often quantified using continuous relative phase (CRP), captures the temporal relationships between joints during cyclic movements [5, 6]. Coordination variability indicates adaptability, yet both excessive and insufficient variability may increase injury risk [7, 8]. Maintaining an optimal level of coordination variability is vital for runners to minimize injury risk without compromising performance [7, 9, 10]. Factors such as aging, gender, injury status, skill level, and running technique influence coordination [11–13]. While treadmill running has dominated research, its controlled conditions limit natural variability, potentially underestimating real‐world adaptations [9, 13–15].

The relationship between coordination variability and running‐related injuries remains debated. Some studies suggest reduced variability in injured runners, reflecting diminished locomotor complexity [16, 17], whereas others report inconsistent findings influenced by fatigue, experience, and biomechanics [12, 18]. Fatigue from long‐distance running alters mechanics, particularly among novices, but most studies use simulated protocols rather than prolonged running relying on short, simulated fatigue protocols rather than ecologically valid prolonged running [19, 20]. Moreover, coordination variability at anaerobic threshold speeds and across surfaces is not well established [10]. Researchers have highlighted the need for biomechanical studies that incorporate ecological validity, including natural terrain, self‐selected pacing, and fatigue accumulation over longer durations [21, 22]. Advances in wearable technology, such as inertial measurement units (IMUs), allow detailed kinematic assessment beyond laboratory environments, enabling researchers to capture coordination patterns during genuine outdoor running [23, 24]. These tools provide new opportunities to examine coordination and coordination variability under realistic conditions that better reflect runners’ everyday training.

Therefore, the present study aims to compare lower‐limb coordination and coordination variability during prolonged treadmill versus over‐ground running in healthy adults. Using CRP analysis of knee–hip, ankle–knee, and ankle–hip couplings, it was hypothesized that (1) coordination variability would be greater during over‐ground running than treadmill running, and (2) variability would increase with running duration due to the higher adaptive demands and accumulated fatigue characteristic of natural running environments.

## 2. Materials and Methods

### 2.1. Study Design

A crossover design was adopted in which the same participants completed both study phases. A two‐way repeated‐measures mixed‐design analysis of variance (ANOVA) was performed to examine the effects of running duration and surface on coordination and its variability in knee–hip, ankle–knee, and ankle–hip joint couplings in the sagittal plane during long‐distance running.

### 2.2. Participants

Eleven healthy adult recreational runners (nine males; mean age: 40.8 ± 8.9 years; weight: 61.4 ± 8.5 kg; height: 170.2 ± 6.3 cm) were recruited from the university and local community through advertisements and invitations. Inclusion criteria required participants to have no history of musculoskeletal injury. Exclusion criteria included current pain, gait abnormalities, or neurological conditions affecting balance. All participants completed the revised Physical Activity Readiness Questionnaire prior to data collection and provided written informed consent. The Human Research Ethics Committee of the institutional review board approved all study procedures.

### 2.3. Procedure

**Figure 1:**
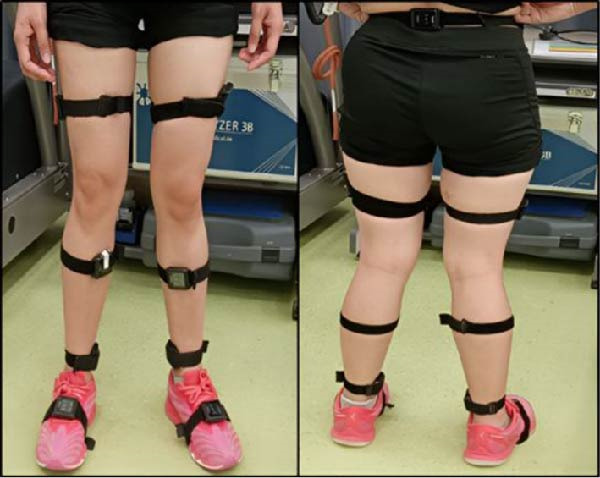
Placement of inertial measurement units on the lower back at the level of L5S1 and two each on bilateral thighs, shank, and foot using elastic straps.

The study was conducted in two phases, performed in random order on separate days. In phase 1, participants ran indoors on a treadmill (GE Marquette 2000, USA) while in phase 2 participants ran outdoors on an all‐weather track. In both phases, subjects ran for 31 min at their preferred speed [25]. Prior to each session, they performed a warm‐up and wore comfortable running attire. Seven IMUs (Opal Movement Monitoring, APDM Inc., Portland, OR, USA) were secured using elastic straps: one on the lower back at the L5–S1 level, and two each on bilateral thighs, shanks, and feet [26–28] (Figure 1).

### 2.4. Data Processing

Raw unfiltered data were collected via Mobility Lab software (MoveoExplorer package, APDM) and processed in MATLAB (MathWorks BV, USA). The middle 30 min of each trial were analyzed to avoid effects of recording initialization and termination. Comparisons were made between the initial 5 min (T1) and final 5 min (T2) of running on each surface. Data were filtered with a second‐order Butterworth low‐pass filter (cutoff: 10 Hz) to remove high‐frequency noise. Heel strike (initial contact) was identified using the peak hip extension of the contralateral leg [29]. Toe‐off was identified at peak knee extension to define stance and swing phases [30]. Each running cycle time series was interpolated to 101 data points to standardize cycle length.

### 2.5. CRP Analysis

**Figure 2:**
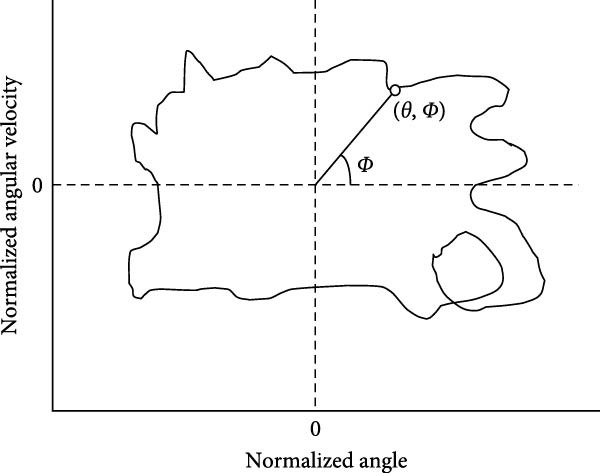
Depiction of phase plot of two joints or segments with phase angle (*Ф*) calculated from each phase plane of normalized angular displacement (*θ*: *x*‐axis) versus normalized angular velocities (*ω*: *y*‐axis).

Kinematic data were analyzed using CRP throughout the gait cycle [31–35]. Figure 2 depicts phase plot of two joints or segments with phase angle (*Ф*) calculated from each phase plane of normalized angular displacement (*θ*: *x*‐axis) versus normalized angular velocities (*ω*: *y*‐axis). Phase angle (*ϕ*) for each joint was constructed by plotting normalized joint angles (*θ*) against normalized angular velocities (*ω*):

ϕ=arctanω/θ.

The CRP for data point *i* was calculated as the difference between the phase angles of the proximal and distal joints:

CRPi=ϕprox,i−ϕdist,i,

where *ϕ*_prox, *i*_ and *ϕ*_dist, *i*_ represent the proximal and distal joint phase angles, respectively [36, 37].

CRP variability (CRPv) was computed as the standard deviation (SD) of CRP values across gait cycles:

CRPv=SDCRPi.

### 2.6. Parameters

a. CRP: CRP describes the relative coordination between joints. A CRP of 0° indicates perfectly in‐phase motion, while 180° indicates perfectly antiphase motion. Intermediate values represent partial synchrony, with lower values closer to in‐phase and higher values closer to antiphase [36, 37]. CRP has been widely applied in clinical and sports biomechanics to quantify coordination patterns and detect impairments [36, 38–41].
b. CRPv: CRPv quantifies the stability of coordination, with lower values reflecting more consistent movement patterns. In this study, three approaches were used [35, 37]:
  • Average CRPv across the entire gait cycle at T1 and T2 for treadmill vs. over‐ground running.
  • Average CRPv during stance and swing phases.
  • Point‐by‐point CRPv across the gait cycle, based on the SD of the first and last five gait cycles at T1 and T2.

CRPv is a sensitive marker of adaptability and has been used to differentiate healthy from injured runners [32, 42, 43].

### 2.7. Data Analysis

Statistical analyses were performed using SPSS version 21 (IBM Corp., Chicago, IL, USA). A two‐way (2 × 2) repeated‐measures mixed‐design ANOVA assessed differences in CRP and CRPv between surfaces (treadmill vs. over‐ground) and time intervals (T1 vs. T2). Significance was set at *p* < 0.05. Post hoc analyses were adjusted using Bonferroni correction.

## 3. Results

**Figure 3:**
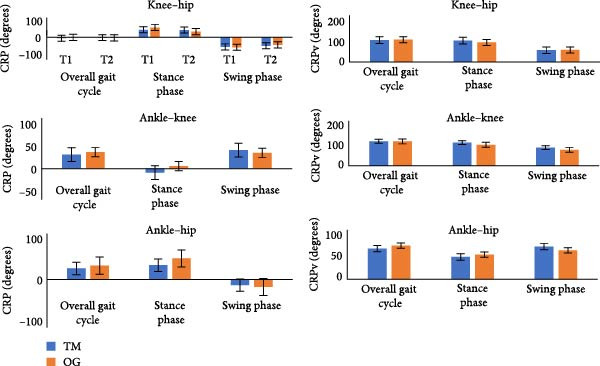
Comparison of coordination (denoted by continuous relative phase angle, CRP) and its variability (CRPv) of knee–hip, ankle–knee and ankle–hip couplings while long‐distance running on both surfaces (treadmill, TM and over‐ground, OG) of running. Values are reported degrees as mean and standard error (SEM). *Note:* There are no significant differences between the two surfaces of running during overall gait cycle, stance, and swing phases.

Mean and SD values of CRP angles and CRPv for the knee–hip, ankle–knee, and ankle–hip joint couplings in sagittal plane motion during treadmill and over‐ground running are summarized in Tables 1 and 2. Across both time intervals, differences in CRP were minimal, and CRPv values were largely comparable across surfaces. Averaged CRPv with standard error for both surfaces is displayed in Figure 3, where similar overall patterns were observed for all couplings. No statistically significant differences were detected between treadmill and over‐ground conditions.

**Table 1:** Description of mean and standard deviation ± SD of coordination (denoted by continuous relative phase angle, CRP) of knee–hip, ankle–knee and ankle–hip couplings while long‐distance running for both durations (T1: 0–5 and T2: 25–30 min) and surfaces (treadmill, TM and over‐ground, OG) of running.

Running surface	Running duration	
	T1	T2
Overall gait cycle		

Knee–hip coupling		
TM	−6.05 ± 14.25	−2.83 ± 14.55
OG	−1.39 ± 14.91	−4.66 ± 17.92
Ankle–knee coupling		
TM	31.99 ± 25.03	30.58 ± 25.90
OG	34.58 ± 23.17	38.85 ± 18.35
Ankle–hip coupling		
TM	25.94 ± 20.07	27.74 ± 21.02
OG	33.19 ± 20.72	34.19 ± 17.37

Stance phase		

Knee–hip coupling^a^		
TM	44.04 ± 26.46	42.83 ± 33.32
OG	58.12 ± 20.94	33.39 ± 40.94
Ankle–knee coupling^a,b^		
TM	−9.21 ± 23.62	**−8.02 ± 35.66**
OG	−6.44 ± 46.14	**17.57** ± **50.70**
Ankle–hip coupling		
TM	34.83 ± 13.51	34.80 ± 11.65
OG	51.67 ± 43.03	50.97 ± 44.36

Swing phase		

Knee–hip coupling		
TM	−58.35 ± 9.00	−51.97 ± 27.55
OG	−60.86 ± 14.08	−45.80 ± 41.65
Ankle–knee coupling		
TM	37.29 ± 23.08	45.69 ± 27.69
OG	41.14 ± 33.14	29.46 ± 40.36
Ankle–Hip coupling^a,b^		
TM	−21.06 ± 19.90	**−6.27 ± 28.94**
OG	−19.71 ± 34.76	**−16.34 ± 34.44**

*Note:* Significantly higher parameters are denoted by bold text.

^a^Interaction effect phase by time *p* < 0.05

^b^Main effect of time, *p* < 0.05

**Table 2:** Description of mean and standard deviation (SD) of coordination variability (denoted by continuous relative phase angle variability, CRPv) of knee–hip, ankle–knee and ankle–hip couplings while long‐distance running for both durations (T1: 0–5 and T2: 25–30 min) and surfaces (treadmill, TM and over‐ground, OG) of running.

Running surface	Running duration	
	T1	T2
Overall gait cycle		

Knee–hip coupling		
TM	107.84 ± 14.00	104.45 ± 18.92
OG	109.41 ± 18.96	105.61 ± 15.39
Ankle–knee coupling		
TM	116.06 ± 18.74	120.29 ± 17.32
OG	116.67 ± 21.60	118.70 ± 12.89
Ankle–hip coupling		
TM	69.60 ± 17.48	78.38 ± 27.68
OG	82.55 ± 31.02	80.17 ± 29.57

Stance phase		

Knee–hip coupling		
TM	107.70 ± 26.50	99.74 ± 24.28
OG	92.45 ± 29.99	96.24 ± 29.81
Ankle–knee coupling		
TM	112.62 ± 29.92	110.78 ± 28.57
OG	97.12 ± 40.94	105.82 ± 35.39
Ankle–hip coupling		
TM	47.94 ± 17.63	59.25 ± 34.06
OG	59.77 ± 25.12	59.60 ± 23.39

Swing phase		

Knee–hip coupling		
TM	52.56 ± 7.63	60.90 ± 16.18
OG	60.31 ± 15.05	56.47 ± 11.59
Ankle–knee coupling		
TM	80.27 ± 41.15	94.71 ± 36.89
OG	76.25 ± 30.67	78.18 ± 28.38
Ankle–hip coupling		
TM	70.95 ± 40.89	87.01 ± 40.86
OG	72.15 ± 34.72	67.76 ± 28.78

Lower limb coordination (CRP): During the overall gait cycle, CRP values did not differ significantly between treadmill and over‐ground conditions or between the initial and final 5 min of running (*p*  > 0.05). In the stance phase, however, ankle–knee coupling showed significantly higher coordination in the final 5 min compared with the initial 5 min (*p*  < 0.05). Simple main effects were also observed in the knee–hip and ankle–knee couplings (*p*  < 0.05). Specifically, for knee–hip coupling, a surface × duration interaction was found during over‐ground running, with coordination significantly higher at T2 compared to T1 (*p*  < 0.05). Conversely, in ankle–knee coupling, a similar interaction indicated that coordination was significantly higher at T1 compared to T2 during over‐ground running (*p*  < 0.05). In the swing phase, ankle–hip coupling demonstrated significantly higher CRP in the final 5 min compared with the initial interval (*p*  < 0.05). A surface × duration interaction was also noted in ankle–hip coupling during treadmill running, with higher coordination observed at T2 (*p*  < 0.05).

Coordination variability (CRPv): For the overall gait cycle, stance, and swing phases, CRPv values did not differ significantly between treadmill and over‐ground conditions or between the two‐time intervals (*p*  > 0.05). These findings indicate stable coordination variability across running surfaces and durations.

**Figure 4:**
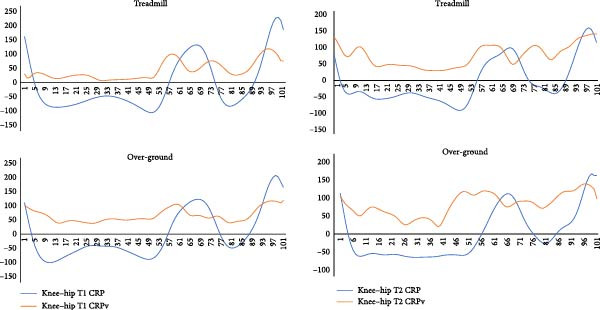
Coordination (denoted by continuous relative phase angle, CRP) and its variability (CRPv) of the knee–hip coupling in the sagittal plane movements averaged over the first (initial, T1) and the last (final, T2) five gait cycles while treadmill and over‐ground running. *Note:* Whole gait cycle is normalized to 101 data points. CRPv represents between subject variability at each point of stride for entire gait cycle. The *x*‐axis represents the gait cycle (%) and the *y*‐axis represents CRP/CRPv (°).

**Figure 5:**
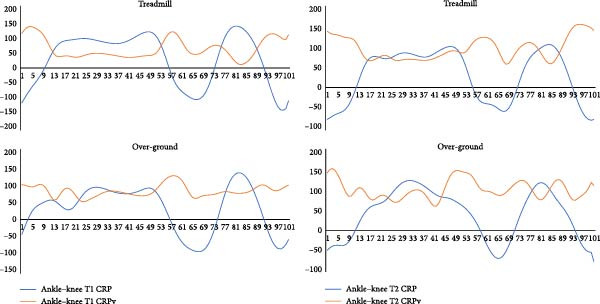
Coordination (denoted by continuous relative phase angle, CRP) and its variability (CRPv) of the ankle–knee coupling in the sagittal plane movements averaged over the first (initial, T1) and the last (final, T2) five gait cycles while treadmill and over‐ground running. *Note:* Whole gait cycle is normalized to 101 data points. CRPv represents between subject variability at each point of stride for entire gait cycle. The *x*‐axis represents the gait cycle (%) and the *y*‐axis represents CRP/CRPv (°).

**Figure 6:**
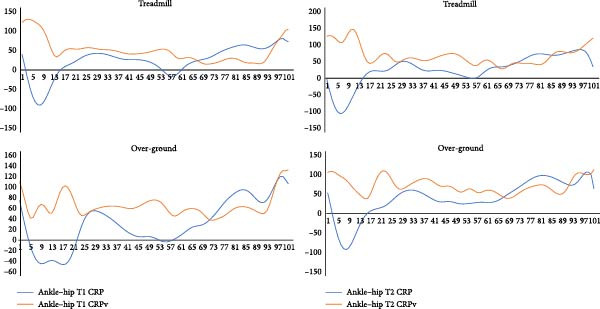
Coordination (denoted by continuous relative phase angle, CRP) and its variability (CRPv) of the ankle–hip coupling in the sagittal plane movements averaged over the first (initial, T1) and the last (final, T2) five gait cycles while treadmill and over‐ground running. *Note:* Whole gait cycle is normalized to 101 data points (*y*‐axis). CRPv represents between subject variability at each point of stride for entire gait cycle. The *x*‐axis represents the gait cycle (%) and the *y*‐axis represents CRP/CRPv (°).

Continuous analysis of coordination: Figures 4–6 present the continuous CRP and CRPv profiles of knee–hip, ankle–knee, and ankle–hip couplings. During treadmill running, knee–hip CRPv remained stable until mid‐cycle and then increased toward the end of the cycle, reaching its peak near toe‐off. A similar trend was observed in over‐ground running, although with greater irregularity in the latter half of the gait cycle. For the ankle–knee coupling, CRPv decreased immediately after heel strike, remained stable through mid‐cycle, and then rose again toward toe‐off. The ankle–hip coupling showed reduced CRPv following heel strike, which remained consistently low throughout most of the cycle before increasing again just prior to toe‐off. Across couplings, treadmill and over‐ground running exhibited broadly comparable patterns, though variability was more irregular in the over‐ground condition. While differences between surfaces were apparent visually, they did not reach statistical significance, likely due to wide confidence intervals.

## 4. Discussion

This study investigated lower limb joint coordination, measured by CRP angles and their variability (CRPv), during long‐distance treadmill and over‐ground running. By comparing the initial and final stages of a 30‐min run, it provides novel insights into stride‐to‐stride variability in coordination outside of a controlled laboratory environment. Across the full gait cycle, no significant differences were observed in CRP or CRPv between treadmill and over‐ground running, or between the start and end of the run. However, phase‐specific changes emerged. In the stance phase, ankle–knee coupling showed higher coordination in the final 5 min, while knee–hip coupling during over‐ground running increased toward the end of the run. Conversely, ankle–knee coupling was greater in the initial stage of over‐ground running. In the swing phase, ankle–hip coordination increased in the final 5 min, with treadmill running also showing higher ankle–knee coupling toward the end. These findings suggest that while overall variability did not differ significantly, joint‐specific adaptations in coordination occurred depending on phase, duration, and running surface.

Increased joint coupling indicates stronger synchronization, potentially supporting efficiency and stability, whereas variability reflects adaptability to changing demands [44]. According to dynamic systems theory, coordination reflects self‐organization of multiple interacting subsystems Kelso [45]. While CRP has been widely applied in walking and clinical gait analysis [40], fewer studies have examined running, and most have focused on footwear, speed, injury, or demographic influences [32, 46, 47]. This study is among the first to use IMUs to assess coordination during prolonged running in natural settings, allowing analysis of ecological motor behavior.

Fatigue is an important factor in running biomechanics and injury risk [48, 49]. Our findings of increased CRP values in ankle–knee, knee–hip, and ankle–hip couplings in the later stages of running are consistent with reports that fatigue alters coordination and may reduce efficiency [12, 50, 51]. Although treadmill‐based studies have shown surface‐related differences in CRP and CRPv [42, 52], the present study found no main effect of surface, but identified surface–duration interactions, suggesting runners dynamically adapt coordination strategies in response to environmental demands [14, 53].

Interpretation of increased CRP values should be phase‐specific. In stance, higher ankle–knee and knee–hip CRP values may reflect less synchronous movement, possibly due to fatigue‐related compensations that affect stability and energy use. In swing, greater ankle–hip and ankle–knee CRP may indicate altered timing of limb advancement, potentially compromising clearance and propulsion. Thus, continuous monitoring of CRP across phases provides richer insights than cycle‐averaged values.

While most prior studies examined stance or whole gait cycles [12, 32, 37, 54], our findings highlight the need to consider both stance and swing, given their distinct biomechanical functions as closed‐ versus open‐chain phases. CRPv was observed to peak at stance‐to‐swing transitions, consistent with earlier findings [12, 55]. Such transient peaks may reflect instability or higher coordinative demands, and suggest that average CRPv values may mask important within‐cycle adaptations.

Running speed is another key factor. Although participants ran at preferred speed to ensure natural gait, prior work shows CRPv decreases at higher speeds, while CRP remains stable [56]. Thus, the absence of CRP differences here may reflect stability within the comfortable speed range. As proposed by dynamic systems theory, coordination adapts to both local (e.g., fatigue, speed) and global (e.g., surface) constraints to maintain functional equilibrium [45, 57]. Finally, the broader application of CRP measures extends beyond performance monitoring. Increased CRP has been linked to gait abnormalities in neurological disorders, rehabilitation outcomes, injury prediction, motor learning in children, and fall risk in older adults. Thus, stride‐to‐stride CRP monitoring may offer translational value in both clinical and athletic populations.

### 4.1. Limitations

This study has several limitations. Participants ran at preferred speed, which, while ensuring ecological gait, may have introduced variability compared to fixed‐speed protocols [42]. Over‐ground speed is also harder to control than treadmill speed [18, 58, 59]. Future research should explore how systematically varying running speeds influence CRP and CRPv across surfaces. The duration of 30 min, rather than a fixed distance, was chosen to examine fatigue‐related changes; however, individual fatigue thresholds may differ, especially for novice versus experienced runners. Factors such as gender, experience, and fatigue response should be explored in future studies. This study focused only on sagittal plane motions in healthy participants, limiting generalizability. Sensor drift and placement stability are challenges in long‐duration IMU studies, and the small sample size increases risk of type II error. Future research should include larger, more diverse cohorts, examine motions in additional planes, and extend to populations with locomotor impairments.

## 5. Conclusion

This study demonstrates that long‐distance running duration and surface primarily influence lower limb coordination patterns, whereas coordination variability remains largely unaffected. Both fatigue and surface‐specific demands emerge as key determinants of joint synchronization and adaptability, underscoring their importance for training, performance, and rehabilitation contexts. By examining stride‐to‐stride coordination in natural environments using IMUs, the study offers novel insights beyond traditional laboratory settings. These findings highlight the relevance of phase‐specific coordination changes during running and their potential implications for injury prevention, performance optimization, and rehabilitation strategies. Future research should extend this work by exploring different populations, speeds, and movement planes to further clarify how environmental and task‐related factors shape gait organization and control.

## Ethics Statement

Participants were informed about all the procedures adopted in the study to obtain their informed consent before data collection. The ethical approval for this study was granted by the Human Research Ethics Committee of The Education University of Hong Kong (Ref. No. 2022‐2023‐0044).

## Consent

The authors have nothing to report.

## Disclosure

The authors reviewed and approved all content.

## Conflicts of Interest

The authors declare no conflicts of interest.

## Author Contributions

Conceptualization: Zaheen Ahmed Iqbal and Daniel Hung‐Kay Chow. Data curation: Zaheen Ahmed Iqbal and Indy Man Kit Ho. Formal analysis: Indy Man Kit Ho. Funding acquisition: Daniel Hung‐Kay Chow. Methodology: Zaheen Ahmed Iqbal, Daniel Hung‐Kay Chow, and Indy Man Kit Ho. Supervision: Daniel Hung‐Kay Chow. Writing – original draft: Zaheen Ahmed Iqbal. Writing – review and editing: Zaheen Ahmed Iqbal, Daniel Hung‐Kay Chow, and Indy Man Kit Ho.

## Funding

No funding was received for this manuscript.

## Data Availability

The data that support the findings of this study are available upon request from the corresponding author. The data are not publicly available due to privacy or ethical restrictions.
